# Clathrin adaptor GGA1 modulates myogenesis of C2C12 myoblasts

**DOI:** 10.1371/journal.pone.0207533

**Published:** 2018-11-15

**Authors:** Mari Isobe, Sachiko Lee, Satoshi Waguri, Satoshi Kametaka

**Affiliations:** 1 Department of Physical and Occupational Therapy, Graduate School of Medicine, Nagoya University, Nagoya, Aichi, Japan; 2 Department of Anatomy and Histology, Fukushima Medical University, Fukushima, Fukushima, Japan; Tohoku University, JAPAN

## Abstract

During myogenesis, myogenic stem cells undergo several sequential events, including cell division, migration, and cell-cell fusion, leading to the formation of multinuclear myotubes, which are the precursors of myofibers. To understand the molecular mechanisms underlying these complex processes, an RNA interference-based gene depletion approach was used. Golgi associated, gamma adaptin ear containing, ARF binding protein 1 (GGA1), a Golgi-resident monomeric clathrin adaptor, was found to be required for the process of myotube formation in C2C12 cells, a cultured murine myoblast cell line. *Gga1* mRNA expression was upregulated during myogenesis, and *Gga1* depletion prevented the formation of large multi-nucleated myotubes. Moreover, inhibition of lysosomal proteases in *Gga1* knockdown myoblasts increased the amount of insulin receptor, suggesting that GGA1 is involved in the cell surface expression and sorting of the insulin receptor. These results suggested that GGA1 plays a significant role in the formation and maturation of myotubes by targeting the insulin receptor to the cell surface to establish functionally mature myofibers.

## Introduction

Skeletal muscle tissue has essential roles within the body, such as movement, metabolism, glycopexis, and thermoregulation [1]. During muscle development, or muscle repair after damage, muscle satellite cells have crucial roles in the generation of muscle fibers. First, quiescent satellite cells are activated to become myoblasts and their number increase. Second, the differentiated myoblasts migrate into the damaged areas within the muscle. Third, multi-nucleated myotubes are formed through myoblast-to-myoblast or myoblast-to-myotube cell fusion [1]. The formation, maintenance, and growth of healthy skeletal muscle tissue are dependent upon these elementary steps.

During myogenic differentiation, myoblast cells undergo drastic changes in cell shape as a result of cell-to-cell fusion, becoming large, multi-nucleated myotubes that are the functional precursors of skeletal muscle cells. In the course of this differentiation, the secretion of several growth factors [2,3] and the cell surface expression of the fusion machinery are essential for proper muscle generation [4,5]. Therefore, the intracellular protein trafficking system is thought to play a significant role in the stage-specific protein secretion and sorting of several plasma membrane proteins required for myogenesis.

Protein sorting at post-Golgi organelles requires the formation of carrier vesicles, such as clathrin-coated vesicles. A group of proteins termed clathrin adaptors is involved in the recognition of the cargo molecules and the physical formation of the membrane-bound clathrin-coated vesicles from the *trans-*Golgi network (TGN) and endosomes. Adaptor protein complex-1 (AP-1) and monomeric adaptor Golgi associated, gamma-adaptin ear containing, ARF binding proteins (GGA1, 2, and 3 in vertebrates) have been well studied in cultured cells and in animal models [6–10].

GGAs share some structural and functional features. All GGAs comprise three globular domains: VHS (Vps27/Hrs/STAM), GAT (GGA and Tom1), and GAE (Gamma-adaptin ear), with the last two domains being connected by a relatively unstructured hinge region. Although the VHS domains of all the GGAs possess similar binding capacity to the (D/E)xxLL-type sorting signal (where D is aspartate, E is glutamate, x is any amino acid, and L is leucine) of several membrane proteins *in vitro* [7], it is also believed that each GGA has its specific interactors. For example, the GAT domains of GGA1 and GGA3 have higher affinity for ubiquitin compared with that of GGA2 [11]. Recently, Uemura et al. showed that p56, an accessory protein of GGAs, is localized at the TGN in a GGA1-dependent manner [12,13]. In addition, while single knock-out (KO) of *Gga1* or *Gga3* caused no obvious phenotypes in mice, the *Gga1/Gga3* double KO or single *Gga2* KO mice were embryonic lethal [9,10]. These results strongly suggested that each GGA has specific physiological roles *in vivo*. However, the detailed physiological functions of GGA proteins remain to be determined.

GGAs have been suggested to be involved in the formation of insulin-responsible vesicles that contain glucose transporter type 4 (GLUT4), insulin-responsive aminopeptidase (IRAP), vesicle associated membrane protein 2 (VAMP2), and adiponectin in adipose tissues [14,15]. Although skeletal muscle is a major tissue responsible for the insulin-dependent uptake of blood glucose *in vivo*, the physiological function of GGA in skeletal muscle remains largely unknown.

In this study, using the cultured mouse myoblast cell line, C2C12, we found that GGA1 is specifically involved in the formation of mature large myotubes. Our data suggested that GGA1 has a pivotal role in the expression of the insulin receptor at the cell surface and in the downstream signaling required to establish mature myotubes.

## Materials and methods

### Cell culture and short interfering RNA-mediated knockdown of *Gga1* in C2C12 cells

C2C12 cells were purchased from ATCC (#CRL-1772) and cultured in growth medium containing Dulbecco’s modified Eagle’s medium (DMEM) (Wako, Osaka, Japan) with 15% fetal bovine serum and 1% penicillin-streptomycin (growth medium). Muscle differentiation of C2C12 cells was induced by changing the medium to DMEM supplemented with 2% fetal bovine serum and 1% penicillin-streptomycin (differentiation medium) for 4 days. To knock down *Gga* gene expression, C2C12 cells were transfected with 20 nM of gene-specific small interfering RNA (siRNA, ON-TARGETplus SMART pool, Dharmacon) using the Lipofectamine RNAiMAX reagent (Thermo Fisher Scientific, Waltham, MA, USA). For control experiments, cells were transfected with non-targeted siRNA (Sigma, St Louis, MO, USA). To measure insulin-dependent glucose uptake, cells were shifted to a serum-free medium for 13 h and treated with Krebs-Ringer-Phosphate-HEPES buffer containing 2% (w/v) bovine serum albumin (BSA) and 1 mM insulin for 5 min at 37 °C. All chemical reagents were purchased from Wako (Japan) unless described.

For the protease inhibition experiment, C2C12 cells were differentiated for 4 days and treated with lysosomal protease inhibitors (0.25 mg/ml leupeptin, 10 mg/ml pepstatin A, and 10 mg/ml E64d) or proteasome inhibitor MG132 at 5 mM in differentiation medium for 18 hours.

### Cloning and expression of mouse GGAs and the preparation of anti-mGGA antibodies

Total RNA isolated from MEF cells was used for cDNA synthesis, and PCR amplification of mouse Gga1 (*mGga1*) and *Gga2* (*mGga2*) and was carried out as described elsewhere [16]. The amplified full-length open reading frames were cloned into pCold-I vector (Takara, Kyoto, Japan) at the *Xho*I—*Xba*I sites. The expression of the recombinant His_6_-mGGAs was induced by cold shift at 15 °C and with 0.5 mM isopropyl β-D-1-thiogalactopyranoside for 24 h, following the manufacturer’s protocol. The His_6_-mGGAs were captured using a Talon metal affinity matrix (GE Healthcare, Chicago, IL, USA) and eluted with 250 mM imidazole, followed by dialysis. The purified recombinant mGGA proteins were used for immunization of rabbits (MBL, Nagoya, Japan) and the obtained antisera were further subjected to affinity purification using the recombinant protein coupled to N-hydroxysuccinimide-activated agarose beads (Thermo Fisher Scientific). The antibodies were checked for their specificity by immunoblotting using cell lysate from C2C12 cells treated with various siRNAs.

### RNA isolation and quantitative real-time PCR

RNA was isolated from C2C12 cells using the RNAiso reagent (Takara) according to the manufacturer’s protocols. Synthesis of cDNA from the RNA samples was performed using PrimeScript 1st Strand cDNA Synthesis Kit (Takara). Real-time PCR amplification was carried out using Thunderbird SYBR qPCR Mix (TOYOBO, Osaka, Japan) on the StepOne Plus (Thermo Fisher Sciences) thermal cycler, according to the manufacturer’s instructions. In brief, real-time PCR was performed using the following cycles: 95°C for 10 min, followed by 40 cycles of 95 °C for 15 s and 60 °C for 1 min for 40 cycles. At the conclusion of the 40 cycles, melting curve analysis was performed to confirm specific amplification. The StepOne software v2.2.2 (Thermo Fisher Sciences) was used for DDCT (cycle threshold) analysis of the data of the real-time PCR [17]. The PCR primer sets used for the real-time PCR amplification are following: *Gga1* (F: CGTCCCCAAGGTCATGAAGGT; R: TGGGGGTTAGCAAGGAGCAG), *MyoD* (myogenic differentiation 1) (F: CCCCGGCGGCAGAATGGCTACG; R: GGTCTGGGTTCCCTGTTCTGTGT), *Myog* (Myogenin) (F: CAACCAGGAGGAGCGCGATCTCCG; R: AGGCGCTGTGGGAGTTGCATTCACT), *Myh3* (myosin heavy chain 3) (F: ATGAGTAGCGACACCGAGATG; R: ACAAAGCAGTAGGTTTTGGCAT), *Insr* (insulin receptor) (F: CCCAGAAAAACCTCTTCAGGCA; R: TGGCTGTCACATTCCCCACC), and *Actb* (beta actin) (F: AAACATCCCCCAAAGTTCTAC; R: GAGGGACTTCCTGTAACCACT).

### Indirect immunofluorescent microscopy

Cells were cultured on round cover slips coated with Matrigel (Corning, New York, NY, USA), diluted with DMEM. Before and after myotube formation, cells were fixed with 4% paraformaldehyde or ice-cold methanol for 10 min. Next, cells were permeabilized and blocked with phosphate-buffered saline (PBS) containing 1 mg/ml BSA and 0.1% Triton X-100 for 15 min at 25 °C. The cells were then incubated with primary antibodies for 1 h at 25 °C or overnight at 4 °C. Cells were washed in PBS with 0.1% Triton X-100 three times, incubated with Alexa Fluor-conjugated secondary antibodies (Invitrogen, Carlsbad, CA, USA), diluted 1:400 in PBS, counterstained with Hoechst 33342, and mounted. The primary antibodies used were as follows: anti-GGA1 or 2 (this study), anti-Golgin subfamily A member 2 (GM130), anti-Ras-related protein rab-4A (RAB4), anti-clathrin heavy chain (CHC), and anti-adaptor related protein complex 1 subunit gamma 1 (AP1G1) antibodies were from BD Biosciences (San Jose, CA, USA), whereas anti-lysosomal associated membrane protein 1 (LAMP1; 1D4B) rat monoclonal antibody, anti-MYH3, and anti-beta actin antibodies were purchased from Santa Cruz (Dallas, TX, USA). The immunofluorescent images were analyzed by BZ-9000 (Keyence, Osaka, Japan) and LSM5 Pascal laser scanning confocal microscopy (Zeiss, Jena, Germany).

### Assessment of cell fusion ability and myotube formation

The fusion index was defined as the percentage of the number of nuclei contained in multi-nucleated myotubes to the total number of nuclei contained within the given field. For each experimental condition we selected six consecutive fields of view. Average fusion indices and standard errors were calculated, and the statistical analysis was conducted using Welch’s *t*-test. *P*-values of less than 0.01 were considered as statistically significant. To assess the level of MYH3, myotubes were stained with anti-MYH3 antibody followed by Alexa488-conjugated anti-Rabbit IgG (Thermo Fisher Scientific). The fluorescent images were captured by BZ-9000 (KEYENCE). The MYH3-positive, multinucleated myotubes were selected and the intensity of MYH3 was quantified using ImageJ software. To obtain the averaged width of myotubes, the area of MYH3-positive myotubes was divided by the longitudinal length.

### SDS-PAGE and immunoblotting analysis

Cells were lysed in cell lysis buffer (PBS, 1% Triton X-100, and 1× Protease Inhibitor Cocktail (Roche)). The samples were then centrifuged at 20,600 × *g* for 20 min at 4 °C and the supernatant was recovered. After a protein assay was performed, samples were subjected to sodium dodecylsulfate polyacrylamide gel electrophoresis (SDS-PAGE) (10 mg/lane). Proteins were transferred to polyvinylidene fluoride membranes (Millipore, Burlington, USA) in transfer buffer (25 mM Tris, 19.2 mM glycine, 0.1% SDS, and 20% methanol). After blocking with 10% non-fat dry milk or 0.1% (w/v) BSA in PBS-T (PBS with Tween-20), the membranes were incubated overnight at 4 °C with primary antibodies. The primary antibodies used were anti-GGA1, anti-GGA2, anti-MYH3 (Santa Cruz), anti-β-actin (Santa Cruz), anti-insulin receptor (Santa Cruz), anti-insulin like growth factor 1 receptor (IGFIR; Santa Cruz), anti-phospho-AKT serine/threonine kinase 1 (AKT1), and anti-AKT1 (Cell Signaling Technology, Danvers, MA, USA). Membranes were then washed with PBS-T and incubated with horseradish peroxidase-conjugated secondary antibodies (GE Healthcare). Lastly, membranes were washed with PBS-T and detected using the enhanced chemiluminescence system (GE Healthcare) and Ez-Capture ST (ATTO Corp., Tokyo, Japan).

### Measurement of glucose uptake

Glucose uptake was assayed using the 2-deoxy-D-glucose (2-DG) Uptake Measurement kit (Cosmobio, Tokyo, Japan). In brief, differentiated (day 4) C2C12 cells were incubated in serum-free high glucose DMEM for 7 h at 37 °C. After incubation, cells were treated with 1 μM insulin for 18 min at 25 °C, and further incubated with 1 mM 2-DG (Peptide Institute, Osaka, Japan) for 20 min at 37 °C. Cells were recovered and the incorporated 2-DG was quantified by measuring the optical density at 405 nm.

### Surface biotinylation

Growing or differentiated cells cultured in 6-well dishes were washed with ice-cold PBS three times on ice and then incubated with PBS containing 0.5 mM EZ-Link Sulfo- N-hydroxysuccinimide-SS-Biotin (Thermo Fisher Scientific) for 30 min on ice. After labeling, the labeling reagent was removed and replaced with ice-cold Tris-buffered saline (10 mM Tris-HCl (pH 7.5), 154 mM NaCl) for 15 min on ice, to quench the biotinylation reagent. The total lysate was generated by lysing the cells in lysis buffer (PBS containing 1% Triton-X-100 and 1× Protease Inhibitor Cocktail). The total lysate was then incubated with Streptavidin-beads (Sigma Aldrich) and the captured cell surface proteins were subjected to immunoblotting.

### Statistical analysis

All data are presented as mean ± standard deviation and were compiled from at least two independent experiments performed in duplicate. *P* values were obtained using an unpaired Welch’s *t*-test or Student’s *t*-test analysis of variance. Values were considered statistically significant when *P* < 0.05.

## Results

### Preparation of specific antibodies recognizing mouse GGA1 and GGA2

**Fig 1:**
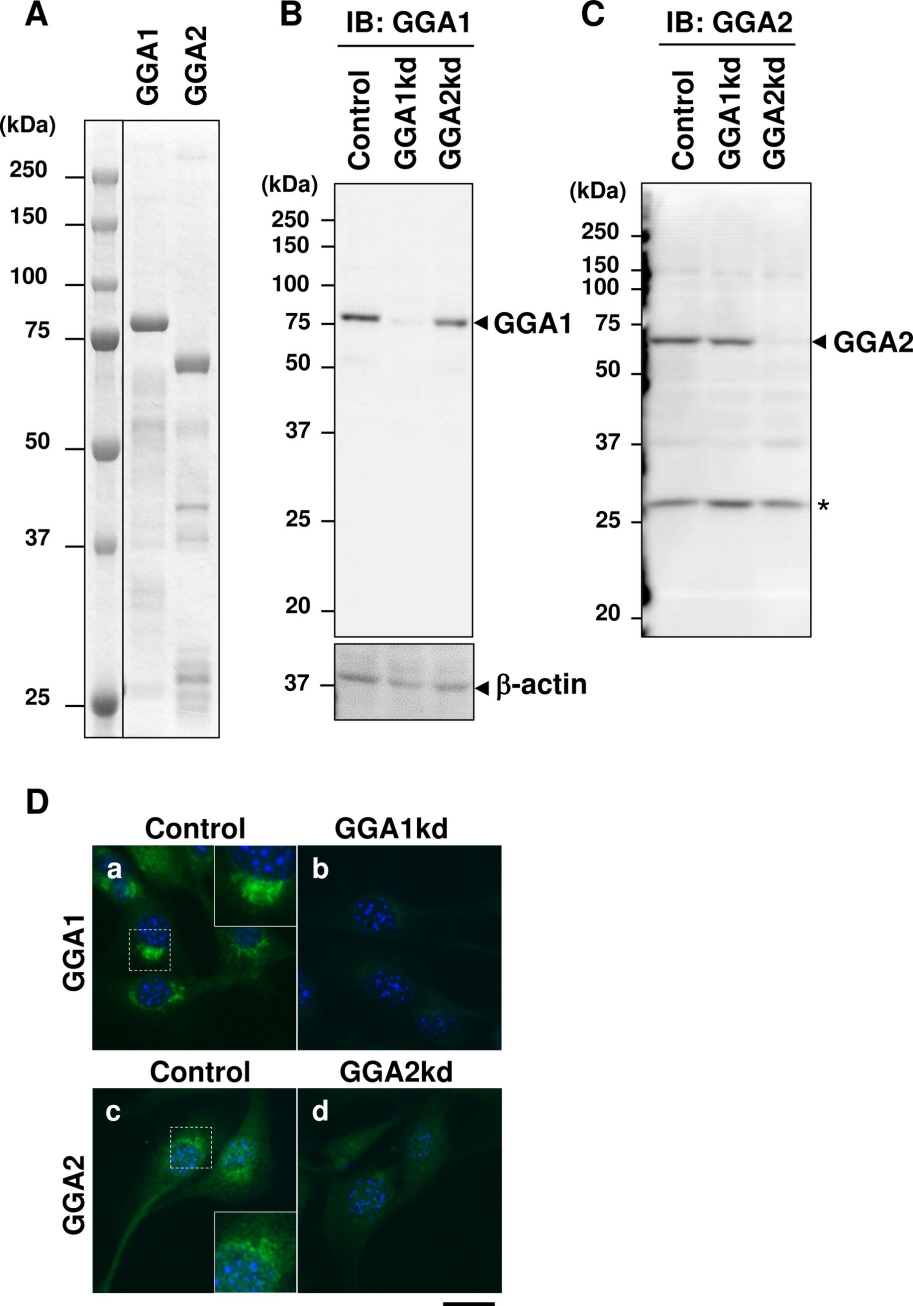
Preparation of specific antibodies to mGGA1 and mGGA2. (**A**) Purified His6-tagged mouse Golgi associated, gamma-adaptin ear containing, ARF binding protein (GGA)1 (mGGA1) and GGA2 (mGGA2) full-length protein expressed in bacteria. Approximately 2 mg of each recombinant protein was subjected to SDS-PAGE and visualized by Coomassie brilliant blue staining. (**B** and **C**) Endogenous GGA1 (**B**) and GGA2 (**C**) were detected using specific antibodies. Lysates from untreated (Con) or GGA1 depleted (GGA1kd), or GGA2 depleted (GGA2kd) C2C12 cells were subjected to immunoblotting with anti-GGA1 (B) or anti-GGA2 (C) antibodies. (**D**) C2C12 cells were treated with control short interfering RNA (siRNA) (a, c) or siRNAs to *Gga1* (b) and *Gga2* (d), and the cells were subjected to immunofluorescence microscopy with anti-GGA1 (a and b: green) and anti-GGA2 (c and d: green) antibodies. Nuclei were counterstained with Hoechst33342 (blue). Scale bar: 20 mm.

To explore the physiological functions of GGAs in myogenesis, effective antibodies were required to detect endogenous GGAs. The commercially available antibodies for human GGAs did not work well to detect mouse GGAs in the mouse myoblast C2C12 cells; therefore, we generated anti-mouse GGA antibodies. The bacterially expressed full-length recombinant mGGA1 and mGGA2 (Fig 1A) were used for immunization of rabbits to raise specific antibodies. The affinity purified anti-mGGA antibodies were assessed for their specificity by immunoblotting using a C2C12 cell lysate (Fig 1B and 1C). The anti-mGGA1 and mGGA2 antibodies produced signals of approximately 81.8 kDa and 67.9 kDa, respectively, in the C2C12 lysate. Moreover, the signals were abrogated when the cells were treated with siRNAs targeting the corresponding gene. These results indicated that the antibodies could successfully detect the endogenous GGA proteins in C2C12 cells. Next, these antibodies were used for indirect immunofluorescence microscopy to check their utility in the morphological analysis. The C2C12 cells treated with siRNAs for *Gga1* or *Gga2* were stained with anti-GGA1 and GGA2 antibodies. In both cases, a perinuclear signal was predominantly observed and the immunoreactivity was reduced by treatment with the siRNAs targeting corresponding genes, indicating that the antibodies specifically recognize the endogenous gene products (Fig 1D).

### GGA1 localizes to the Golgi apparatus and endosomes in differentiating C2C12 cells

**Fig 2:**
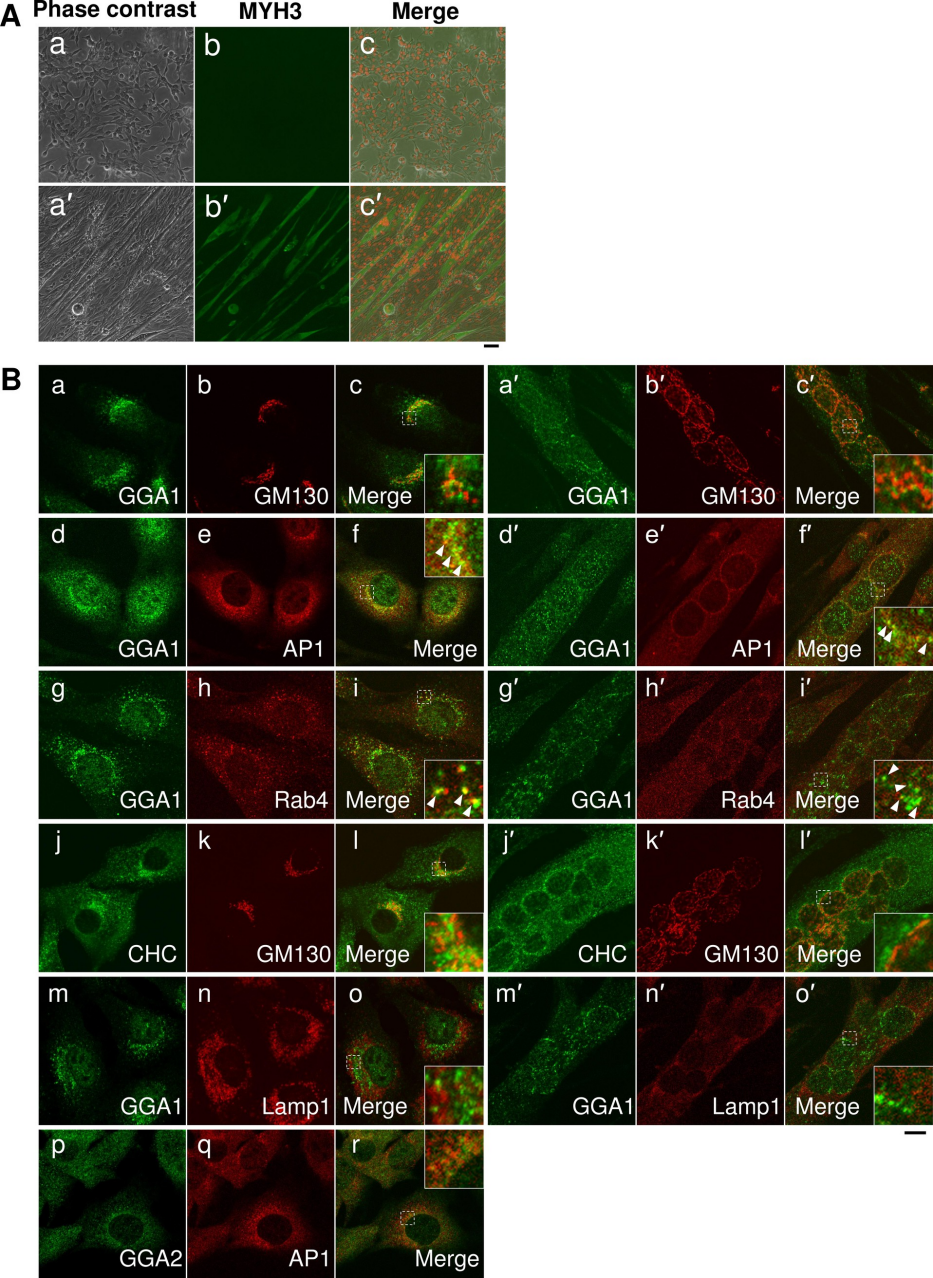
GGA1 is distributed from the Golgi to endosomes in C2C12 cells during myogenesis. (**A**) Myogenic differentiation of C2C12 cultured myoblasts. C2C12 cells cultured in growth medium (day 0) and shifted to differentiation medium for four days (day 4) were stained with anti-myosin heavy chain 3 antibodies. (**B**) C2C12 cells cultured in the growth medium (day 0, a-o) and differentiation medium for four days (day 4, a′-o′) were subjected to immunofluorescent microscopy using anti-golgi associated, gamma adaptin ear containing, ARF binding protein 1 (GGA1) (a, c, d, f, g, i, m, o, a′, c′, d′, f′, g′, i′, m′, and o′), anti-golgin subfamily A member 2 (GM130) (b, k, b′, and k′), anti-adaptor related protein complex 1 subunit gamma 1 (AP1G1) (AP1: e, n, e′, and n′), anti-Ras-related protein rab-4A (RAB4) (h and h′), anti-clathrin heavy chain (CHC) (j and j′), and anti-lysosomal associated membrane protein 1 (LAMP1 (n and n′) antibodies. The cells from day 0 were stained with anti-GGA2 (p and r) and anti-AP1G1(q and r). All the images from anti-GGA1 or GGA2 antibodies are shown in green and those from other antibodies are shown in red. Merged images are indicated. Scale bar: 10 mm.

GGA1 localizes to the TGN and endosomes in many cell types and contributes to the formation of clathrin-coated vesicles, which are required for intracellular transport of secretory and integral membrane proteins [7,18]. Thus, because its intracellular localization in differentiating myoblast cells was unknown, the intracellular localization of GGA1 and other organelle markers in growing or differentiating C2C12 cells was examined using indirect immunofluorescent microscopy with specific antibodies. Muscle differentiation was induced in C2C12 cells by shifting the culture medium to differentiation medium containing a low concentration of serum. Approximately four days after shifting to the differentiation medium, many large myotubes with multiple nuclei, which were positive for the developmental myosin heavy chain 3, MYH3, were observed in wild-type cells (Fig 2A).

GGA1 was localized at the perinuclear region and some peripheral puncta, in both the growing cells (day 0) and the multi-nucleated myotubes formed after four days of differentiation (day 4) (Fig 2B, a and a′). Co-staining with several organelle markers showed that the perinuclear localization was closely associated with GM130, a cis-Golgi marker (Fig 2B, a-c and a′-c′). The GM130-positive Golgi structure was also closely associated with, although it did not completely overlap with, the CHC signals (Fig 2B, j-l and j′-l′). GGA1 was also markedly colocalized with the gamma-subunit of the AP-1 complex at the Golgi area before and after differentiation (Fig 2B, d-f and d′-f′). Moreover, the GGA1-positive vesicular structures at the cell periphery also coincided with the signal for RAB4 (Fig 2B, g-i and g′-i′), which is localized at recycling endosomal compartments, whereas the GGA1 signal was completely segregated from that of LAMP-1, a marker for late endosomes and lysosomes (Fig 2B, m-o and m′-o′). These results indicated that GGA1 was distributed from the Golgi apparatus to the endosomes in C2C12 cells, throughout the differentiation period.

In addition to GGA1, the intracellular localization of GGA2 was also analyzed. GGA2 showed very similar staining patterns to those of GGA1 in C2C12 cells (Fig 2B, p-r). However, as the anti-GGA2 antibody gave less clear results compared with those of GGA1, we obtained only limited results from GGA2 staining of C2C12 cells.

### GGA1 depletion causes a defect in myotube formation of C2C12 cells

**Fig 3:**
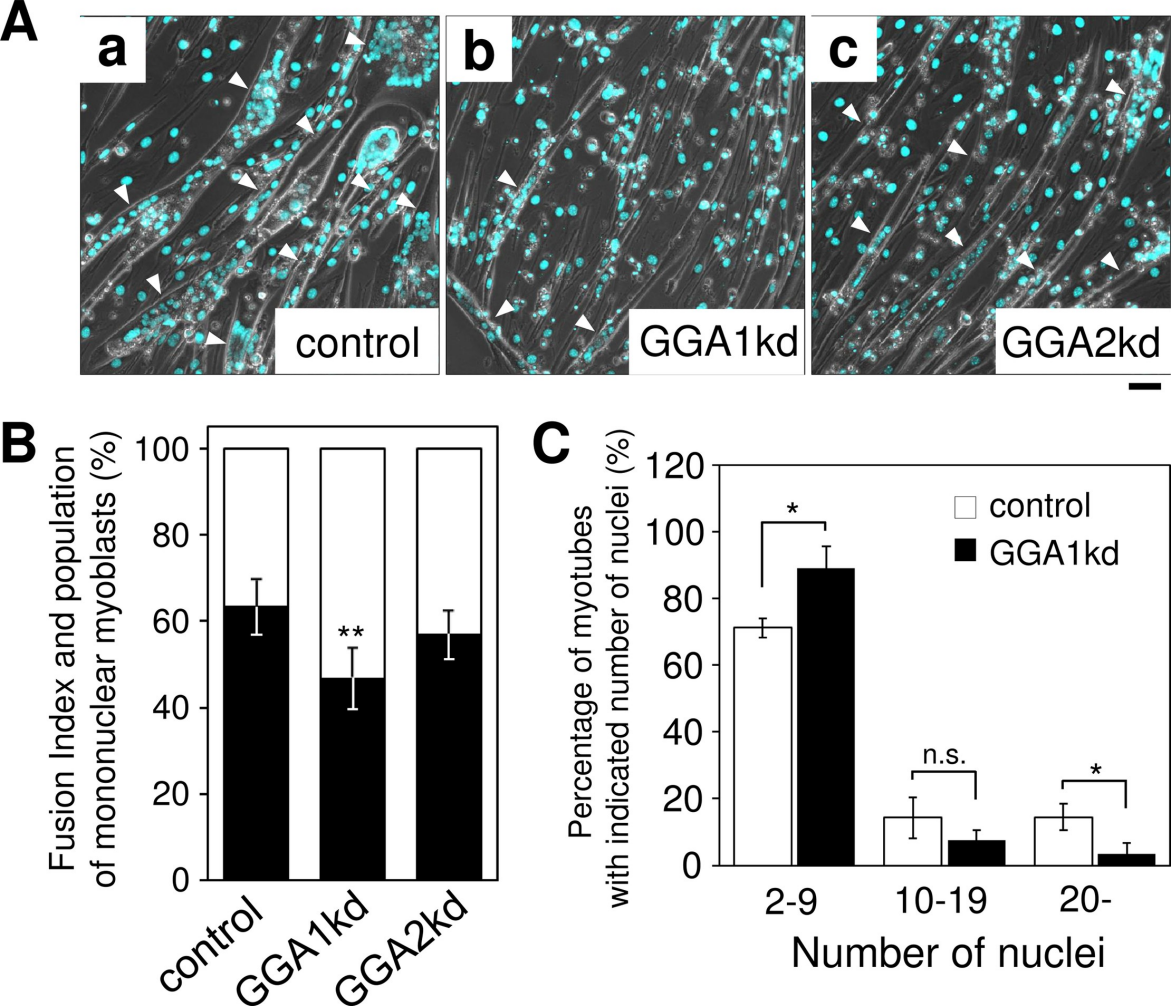
GGA1 is involved in myogenesis of C2C12 cells. (**A**) Knockdown of *Gga1* (Golgi associated, gamma adaptin ear containing, ARF binding protein 1) affects myogenesis of C2C12 cells. Short interfering RNA (siRNA) for non-target (a), *Gga1* (b), and *Gga2* (c) were transfected into C2C12 cells and muscle differentiation was induced for four days. *Gga1*kd cells showed fewer myotubes compared with those formed by the other cells. (**B**) The fusion index in (A) was calculated as described in the Materials and methods. The population of the nuclei in myotubes was indicated by the closed bars and that of unfused mononuclear myoblasts was indicated by the open bars. Error bars indicate SD (n = 6). (**C**) Quantification of the percentage of nuclei in control and *Gga1*kd (siGGA1) myotubes. Error bars indicate SD (n = 3). *. *P* < 0.05, **. *P* < 0.01 by a Welch’s *t*-test. Scale bar: 50 mm.

To assess the physiological function of GGA1 in skeletal muscle differentiation, knockdown of *Gga1* was performed in C2C12 cells. Morphological analysis showed that *Gga1* knockdown (kd) cells showed fewer myotubes compared with control siRNA treated or *Gga2*-depleted cells (Fig 3A and S1 Fig). The results suggested that GGA1, but not GGA2, is possibly involved in myogenesis of C2C12 cells. To quantify the morphological data, the fusion index, which indicates the percentage of nuclei in myotubes as a proportion of the total number of nuclei in a given field, was calculated. The fusion indices of control, *Gga1* kd and *Gga2* kd cells were 63.3%, 46.7% and 56.9%, respectively. The result also indicated that the population of the unfused, mononuclear myoblasts in *Gga1* kd cells was 16.7% higher than that of control cells, whereas no significant increase was observed by knockdown of *Gga2* (Fig 3B). To further analyze which step of myotube formation is impaired by GGA1 depletion, a histogram analysis of the numbers of intracellular nuclei *vs*. the cell number of myotubes was constructed (S2 Fig and Fig 3C). The histogram showed a substantial decrease of 78.6% in the number of large myotubes containing over 20 nuclei in *Gga1*kd cells, and the number of small myotubes with less than ten (2–9) nuclei in Gga1 kd cells significantly increased by 25.1% compared with that of control cells (Fig 3C). These results confirmed that GGA1 is partially involved in the fusion event of myoblasts throughout the myogenesis of C2C12 cells.

### Induction of GGA1 expression during myogenesis of C2C12 cells

**Fig 4:**
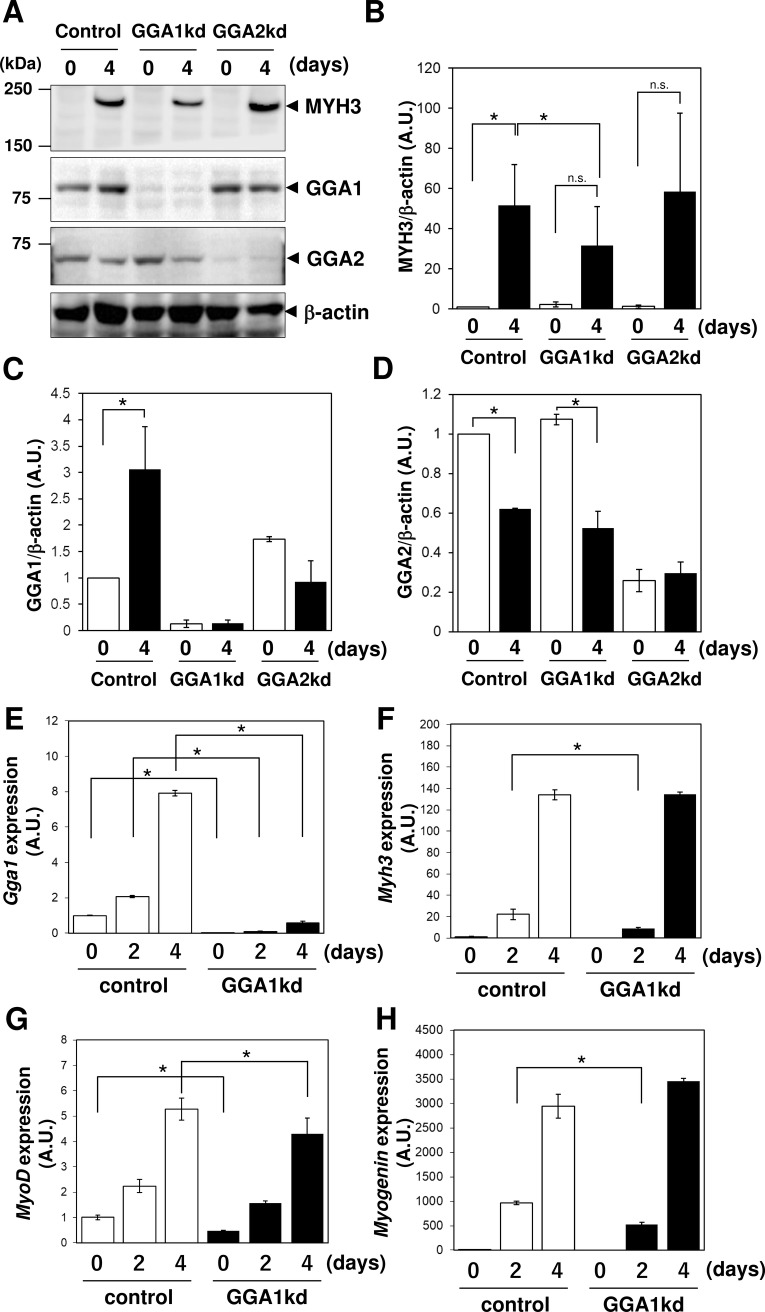
*Gga1* knockdown caused a defect in the expression of MYH3. (**A**) Golgi associated, gamma adaptin ear containing, ARF binding protein (GGA)1, GGA2, and myosin heavy chain 3 (MYH3) expression in control, *Gga1*kd and *Gga2*kd cells before (day 0) and after (day 4) myogenic differentiation were examined by immunoblotting. b-actin was used as a loading control. (**B**) Expression of myogenic genes (*Myod* (myosin D), *Myog* (myogenin), and *Myh3*) and *Gga1* gene at the differentiation periods of 0, 2 and 4 days in the control and *Gga1*kd cells was analyzed by quantitative real-time PCR (qPCR). *Actb* (b-actin) was used as the endogenous control. Error bars indicate SD (n = 3). *. *P* < 0.05 by Welch’s *t*-test.

Silencing of *Gga1* caused a reduction in the number of myotubes, and GGA1 has specific function(s) during myogenesis; therefore, to examine if the expression level of GGA1 is controlled under myogenesis, immunoblotting of GGA1 was performed. GGA1 protein expression was increased by approximately 3-fold during myogenic differentiation, whereas no significant change was observed in GGA2 protein expression. A decreased level of embryonic myosin heavy chain, MYH3, was observed in the *Gga1*kd cells, but not in the *Gga2*kd cells (Fig 4A), which was consistent with the results of the morphological analysis showing the reduced induction of myotubes (Fig 3).

### GGA1 is not required for sequential induction of myogenic genes

During myogenesis, the sequential induction of myogenic transcription factors, including MyoD and Myogenin, leads to the expression of muscle specific genes [19]. The expression of MYH3 was affected by depletion of GGA1, which suggested that GGA1 might be involved in the induction of myogenic genes. To address this point, the expression levels of *Myod* and *Myog* were assessed using qPCR with *Actb* (encoding b-actin) as the control. The expression of *Gga1* mRNA increased by 8-fold during myogenesis for 4 days. However, the transcriptional induction of myogenic genes was not affected by GGA1 depletion, except for a slight reduction of MyoD expression (Fig 4F–4H). These results suggested that the expression of *Gga1* is transcriptionally controlled during myogenesis; however, its expression is not required for the sequential expression cascade of myogenic transcriptional factors.

### Knockdown of *Gga1* caused defects in the formation of mature myotubes

**Fig 5:**
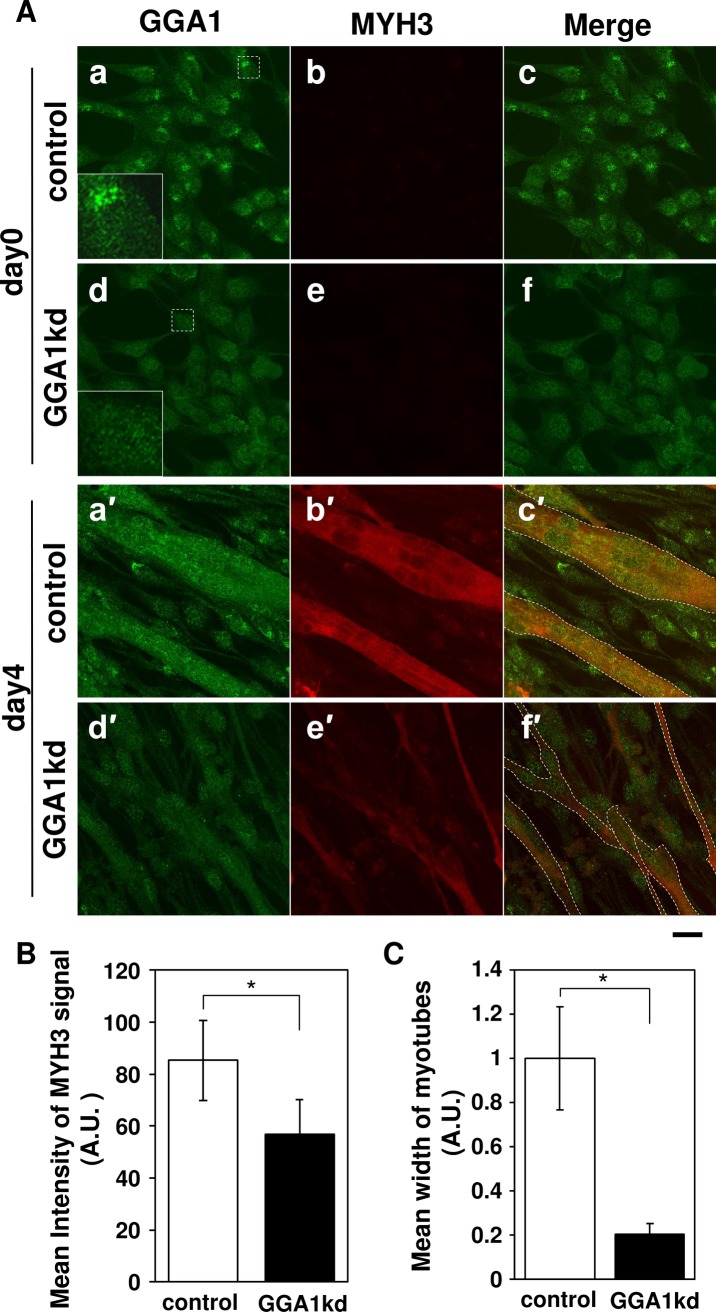
*Gga1* knockdown impaired the formation of large myotubes and expression of MYH3. (**A**) Morphological analysis of Golgi associated, gamma adaptin ear containing, ARF binding protein 1 (GGA1)-depleted myotubes. C2C12 cells transfected with a control short interfering RNA (siRNA) (control: a-c and a′-c′) and *Gga1* siRNA (GGA1kd: d-f and d′-f′) were subjected to immunofluorescence microscopy before (day 0) or after differentiation for four days (day 4) using anti-GGA1 (a, d, a′, and d′: green) and anti-myosin heavy chain 3 (MYH3) (b, e, b′, and e′: red) antibodies. The merged images are indicated in the right column. (**B**) Quantitative analysis of the MYH3 signal in the control and *Gga1*kd myotubes at day 4. The signal density is plotted. Error bars indicate the SD (n = 8). (**C**) Quantitative analysis of the average cell width in the control and *Gga1*kd myotubes at day 4. The value is plotted. Error bars indicate the SD (n = 4). *. *P* < 0.01 by Welch’s *t*-test. Scale bar: 10 mm.

To gain more information about the physiological condition of the myotubes in the *Gga1*kd cells, morphological analysis was carried out. The intensity of MYH3 immunofluorescence in the GGA1-depleted myotubes was approximately 33.2% lower than that in the control cells (Fig 5A, b′ and e′, and 5B), and the average width of the myotubes in the *Gga1*kd cells was reduced by approximately 79.5% (Fig 5C). These data showed that knockdown of *Gga1* resulted in the formation of smaller and thinner myotubes during myogenesis.

### GGA1 deficiency affects insulin-dependent glucose uptake in C2C12 myotubes

**Fig 6:**
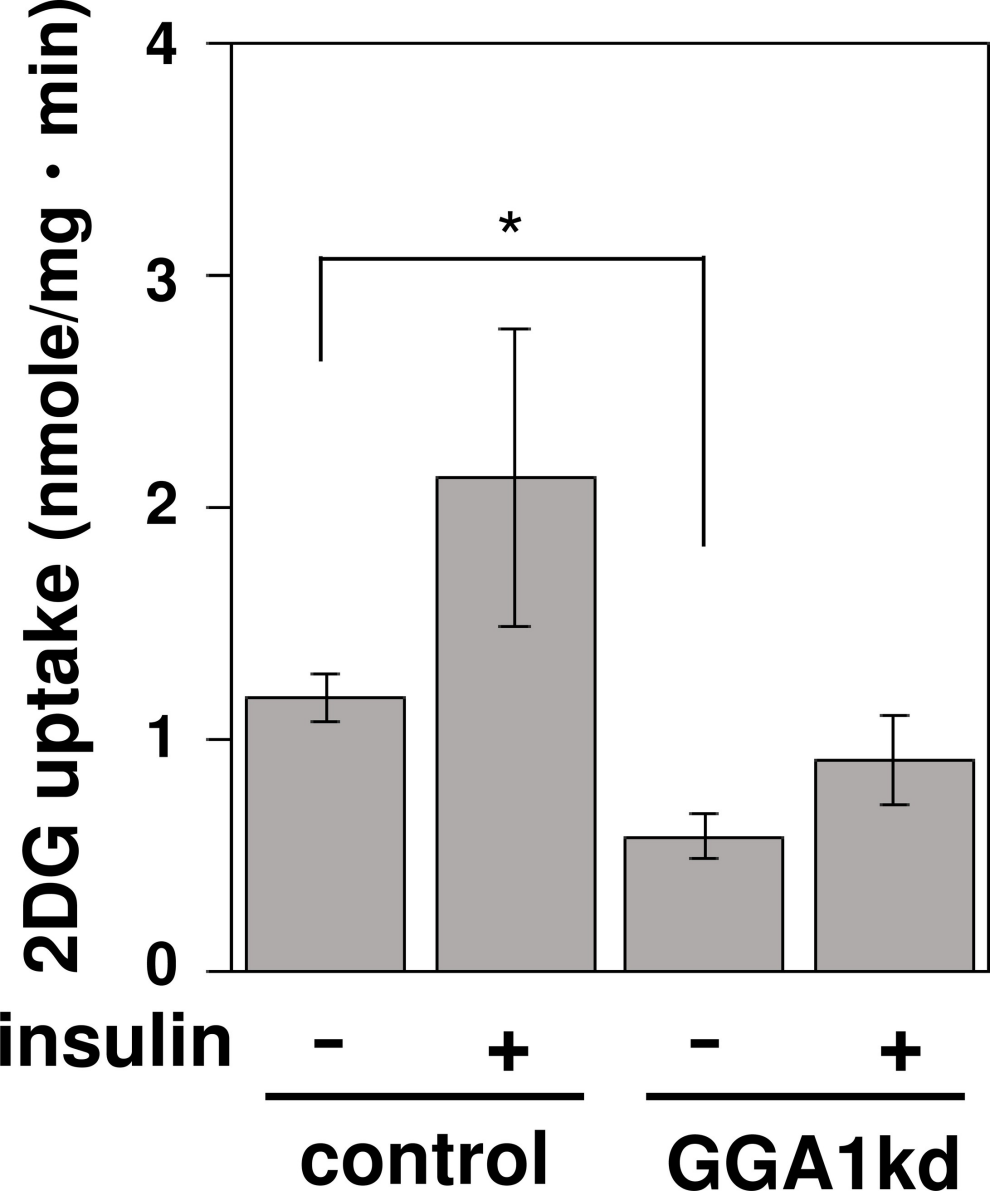
*Gga1* silencing affects the insulin-dependent glucose uptake of myotubes. Control and Golgi associated, gamma adaptin ear containing, ARF binding protein 1 gene knockdown (*Gga1*kd) myotubes were prepared as in Fig 4, and the uptake of 2-deoxy-D-glucose (2-DG) in the absence or presence of 1 mM insulin was assayed as described in the Materials and methods. Error bars indicate the SD (n = 3). *. *P* < 0.05 by a Student’s *t*-test.

Mature skeletal muscle has a large capacity to take up and store blood glucose upon insulin stimulation *in vivo*. During maturation of the skeletal muscle, the levels of the molecules that are involved in insulin-dependent glucose uptake increase to acquire the physiological role [20]. To assess whether the *Gga1*kd myotubes have the wild-type glucose uptake function, *Gga1*kd myotubes were examined for their capacity for insulin-dependent glucose uptake. Control and *Gga1*kd myotubes were prepared, and their glucose uptake ability was assayed using 2-DG, a non-metabolizable glucose derivative. As shown in Fig 6, 2-DG uptake was diminished by *Gga1* knockdown under both basal and insulin-stimulated conditions, indicating that the *Gga1*kd cells failed to differentiate into physiologically mature myotubes.

### GGA1 is involved in expression of the insulin receptor

**Fig 7:**
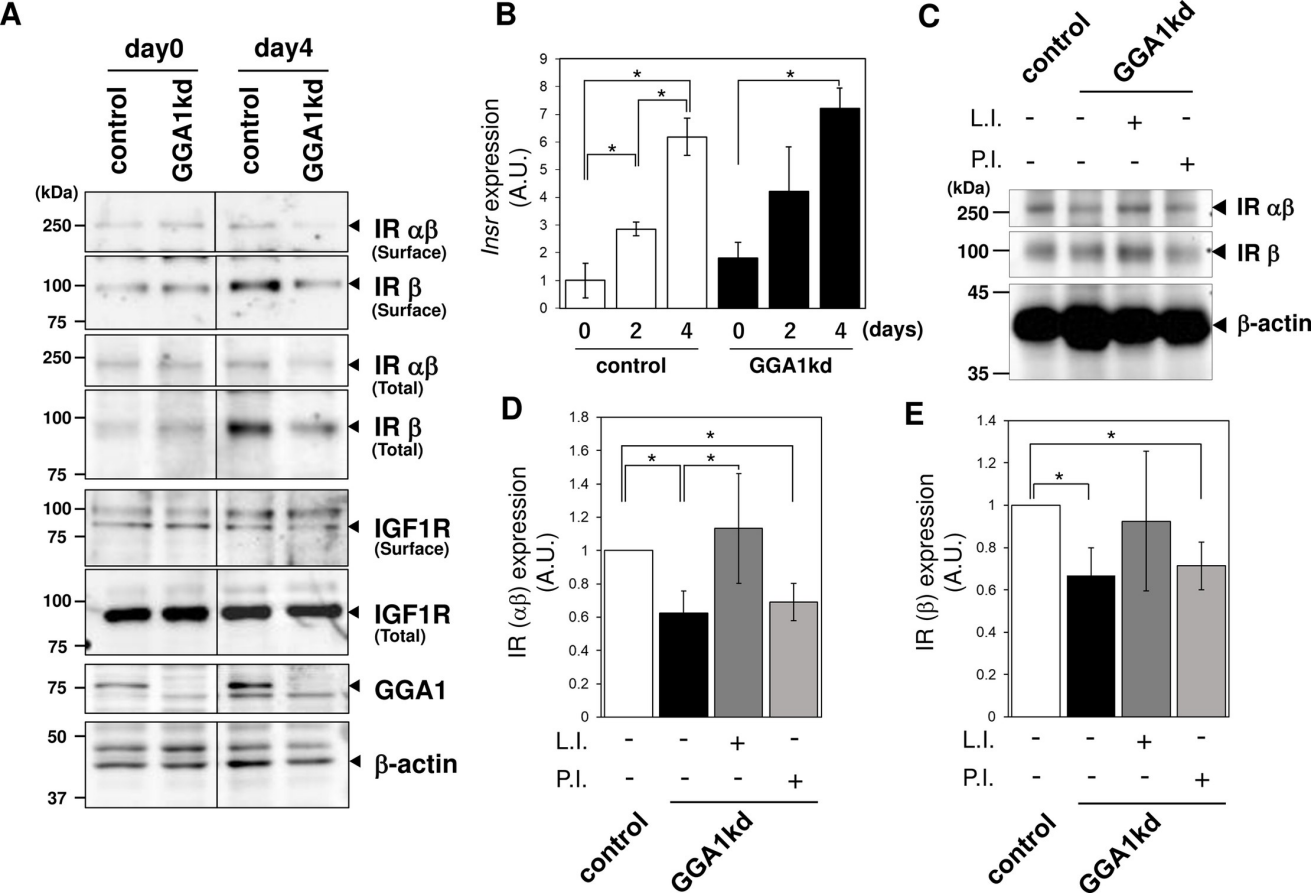
*Gga1* knockdown impaired the expression of the insulin receptor. (A) Total proteins (Total) and the cell surface proteins (Surface) purified using surface biotinylation from growing (day 0) and differentiated (day 4) cells were subjected to immunoblotting for golgi associated, gamma adaptin ear containing, ARF binding protein 1 (GGA1), the insulin receptor (IR), and the insulin-like growth factor 1 receptor (IGF1R). b-actin was used as the loading control. (B) Quantitative real-time PCR (qPCR) for *Insr* (encoding IR) was carried out. (C) Lysosomal inhibitors suppressed the degradation of the IR in *Gga1*kd cells. Myotubes differentiated for 4 days were treated with lysosomal inhibitors (L.I.) or proteasome inhibitor (P.I.) for 18 hours. The total lysates were subjected to immunoblotting for the IR. The signals for the ab precursor (D) and b-chain (E) of the IR were quantified and normalized by the b-actin signal. Error bars indicate the SD (n = 3). *. *P* < 0.05 by Welch’s *t*-test.

Recently, it was shown that GGAs and the related clathrin adaptor AP-1 complex have functions not only in TGN-endosomal/lysosomal membrane trafficking, but also for the surface expression of a series of integral membrane proteins, including Notch and the epidermal growth factor receptor [13,21]. Moreover, Conejo et al. reported that insulin and insulin-like growth factors signaling play significant roles in the maturation of myotubes [22]. Thus, we speculated that the immature *Gga1*kd myotubes with low MYH3 expression had a defect in the cell surface expression of the receptor proteins. To determine the possible changes in the expression levels of surface receptors, immunoblotting for the insulin receptor (IR) and the related receptor insulin-like growth factor receptor (IGF1R) was performed with total cell lysate and the cell surface protein fraction. The expression of the IR (both the b chain and ab precursor) was significantly induced by myogenic differentiation (Fig 7A)[23]. Immunofluorescent microscopy also confirmed that the induction of the IR was mainly observed in the multinucleated myotubes, and a low signal was observed in the surrounding unfused myoblasts (S3 Fig). In addition, the expression of the IR in both fractions was significantly decreased in *Gga1*kd cells, whereas little change was seen in IGF1R levels (Fig 7A). To examine the possibility that GGA1 affects the expression of the IR at the transcriptional level, the expression of *Insr* mRNA (encoding the IR) was quantified by qPCR. There was no significant change in the level of the *Insr* transcript in the *Gga1*kd cells (Fig 7B), suggesting that knockdown of *Gga1* decreased the stability of the IR.

Next, to address the possibility that the IR was degraded in the absence of GGA1, *Gga1kd* myotube cells were treated with inhibitors of lysosomal proteases or the proteasome. The lysosomal inhibitors, but not the proteasomal inhibitor, successfully blocked the decrease in the cellular IR level in *Gga1*kd cells, indicating that the IR was degraded in the lysosomal compartment in the *Gga1*kd cells (Fig 7C–7E). Taken together, these results suggested that GGA1 is specifically required for the stable expression of the IR.

### Insulin signaling is attenuated in GGA1kd myotubes

**Fig 8:**
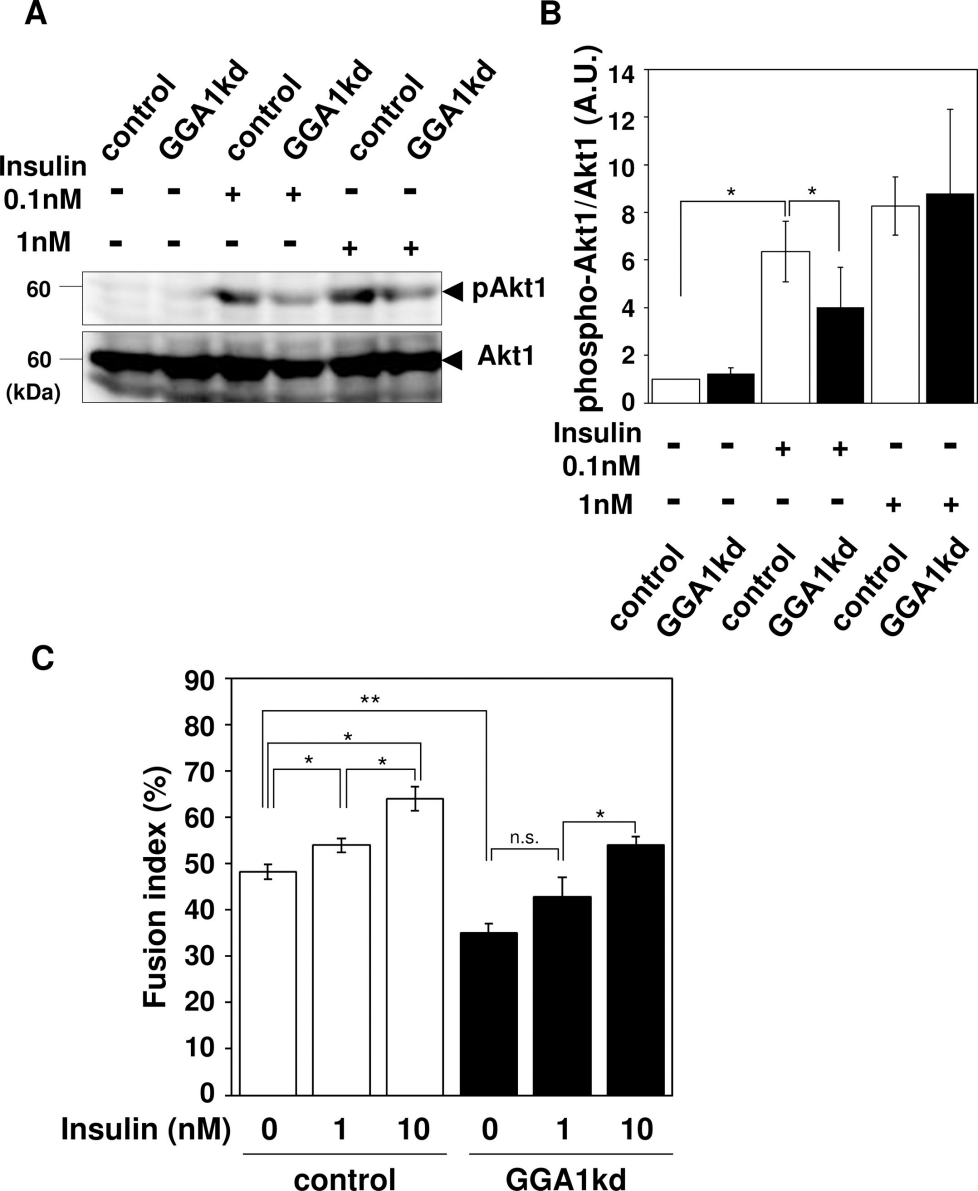
GGA1kd cells exhibit defective insulin response. (**A**) C2C12 cells treated with control and *Gga1* (Golgi associated, gamma adaptin ear containing, ARF binding protein 1) siRNAs were stimulated by 0, 0.1, and 1 nM insulin for 5 min. Insulin signaling was then examined using anti-phospho AKT serine/threonine kinase 1 (AKT1) and anti-pan AKT1-specific antibodies. (**B**) The quantification of the phospho-AKT1/total AKT1 ratio is indicated on the right bar graph. (**C**) Wild-type and *Gga1kd* C2C12 cells were subjected to differentiation in the absence or presence of the indicated concentrations of insulin in the medium for four days. The formation of myotubes was then analyzed. Error bars indicate the SD (n = 3). *. *P* < 0.05, **. *P* < 0.01 by Welch’s *t*-test.

*Gga1*kd caused a reduction in IR surface expression as well as its total cellular level; therefore, we assessed IR downstream signaling to determine whether the signaling pathway was affected by *Gga1*kd at a physiological concentration of insulin. After serum depletion for 24 h, cells were stimulated by 0.1 and 1 nM insulin for 5 min and downstream signaling was examined by assessing AKT1 phosphorylation. Compared with the unstimulated cells, a significant reduction in AKT1 phosphorylation was observed in *Gga1*kd cells, indicating that GGA1 depletion affects insulin signaling (Fig 8A and 8B).

### Insulin-dependent acceleration of myotube formation is affected in GGA1kd myoblasts

A previous study reported that modest insulin stimulation during myogenesis could enhance the formation of myotubes in C2C12 cells [22]. To determine if the insulin-dependent acceleration of myotube formation is also affected by *Gga1* knockdown, C2C12 cells were subjected to myogenic differentiation in the presence of various concentrations of insulin. In the cells treated with the control siRNA, myotube formation increased under stimulation with 2 or 10 nM insulin. In contrast, the *Gga1*kd cells failed to respond to 2 nM insulin, suggesting that GGA1 is required for the enhancement of myotube formation at a physiological concentration of insulin (Fig 8C). At higher insulin concentrations, however, an increase in the fusion index was observed in *Gga1*kd cells, consistent with the significant AKT1 phosphorylation in *Gga1*kd cells observed at higher concentrations of insulin (Fig 8A).

## Discussion

In this study, we found that GGA1 has a crucial role in myogenesis of C2C12 cells. The GGA1-depleted cells showed small and thin myotubes with decreased expression of MYH3. The GGA family proteins are well known for their functions in recognition of cargo proteins that are sorted at the TGN; therefore, we initially speculated that GGA1 was involved in the sorting of fusion machinery to the plasma membrane. Indeed, a recent report showed that GGA2 has a crucial role in the cell surface expression of the epidermal growth factor receptor (EGFR), and the GGA-related clathrin adaptor AP-1 complex is also involved in targeting of the cell surface signaling molecule Notch and the secretion of certain soluble factors [13,21,24,25].

In the present study, morphological analysis indicated that GGA1 depletion caused a significant decrease in the population of myotubes with a large number (> 20) of nuclei, and increase in the number of myotubes with few nuclei. This result strongly suggested that GGA1 depletion affects the fusion process of myoblasts throughout the myogenesis of C2C12 cells. The immaturity of the myotubes in *Gga1*kd cells, however, also implied a significant physiological role of GGA1 in the metabolic maturation of myotubes. Thus, what is the physiological function of GGA1 during myotube formation? The regulation of the expression cascade [26] of myogenic genes *Myod*, *Myog*, and *Myh3* was not significantly affected in *Gga1*kd cells, which suggested that GGA1 is not involved in the transcriptional control of these genes during the myogenic process.

During muscle differentiation of myoblasts, many extracellular factors, such as insulin, insulin-like growth factor I and II, and fibroblast growth factors, play important roles to support the growth of myotubes and myofibers [27,28]. Some of them are secreted from myoblasts and promote their own differentiation in an autocrine manner. In the current study, we found that IR expression was decreased in *Gga1*kd cells (Fig 7). Moreover, the GGA1-depleted cells also showed decreased sensitivity to the insulin stimulation that enhanced myotube formation (Fig 8C). These results suggested that the defect in the formation of large, mature myotubes in *Gga1*kd cells is, at least in part, caused by a reduction of cell surface receptor(s) and the subsequent impaired downstream signaling.

Insulin signaling is involved in skeletal muscle physiology [29]. Mice lacking mammalian target of rapamycin 1 (mTORC1) showed dystrophic skeletal muscle, mild glucose intolerance, and a shortened lifespan [30,31]. Mice lacking both insulin receptor substrate (IRS)1 and IRS2 in skeletal muscle also exhibited a much shorter lifespan than the control mice [32,33], suggesting that insulin action in skeletal muscle has a vital, but unrecognized, role in the control of lifespan, and that mTORC1 may contribute to these effects [34]. The decreased ability to respond to insulin stimulation observed in *Gga1*kd cells implied that GGA1 deficiency could cause diverse defects in insulin signaling *in vivo*.

The reduction of IR levels in *Gga1*kd cells was suppressed by the inhibition of lysosomal proteases, which strongly suggested GGA1 depletion causes the IR to be missorted to the lysosome and degraded. One possible explanation for this result is that GGA1 functions in the recycling compartments that return the IR from the endocytic compartments to the cell surface. Alternatively, GGA1 could be involved in the sorting of IR at the Golgi compartments into the vesicle carriers destined for the plasma membrane. Currently, however, we have not addressed the precise functional site of action of GGA1.

More recently, Uemura and coworkers showed that GGA2 regulates the cell surface expression of EGFR via direct recognition and binding to the cytoplasmic region of EGFR [13]. Notably, the binding region of EGFR did not contain the (D/E)xxLL-type, conventional GGA binding motif. Although it remains unknown whether GGA1 could directly recognize the cytoplasmic domain of IR, which also does not contain a GGA binding motif, our preliminary results from the *in vivo* proximity labeling experiment suggested that GGA1 functions in proximity to the IR *in vivo* (S4 Fig)[35]. Thus, further investigation will clarify how GGA1 contributes to the trafficking of such surface receptors.

Previously, Pessin’s group proposed a model in which GGA proteins promote the loading of GLUT4 into GLUT4 storage vesicles (GSV) [36]. Another GSV resident membrane protein, IRAP, is also loaded into GSVs by GGA, through its di-leucine motif in the cytoplasmic region [37, 38, 39]. Thus GGA1 is likely to play a significant role in GLUT4 loading into GSVs; however, some of the data were obtained using the inhibitory effects of the VHS-GAT domains of GGA1. The VHS-GAT domain of GGAs selectively binds to the GTP form of Arf small GTPases and is presumed to block their actions broadly [40]. Arf1 functions in diverse steps throughout the secretory pathway; therefore, the inhibitory effects of VHS-GAT may not be specific to the GGA-dependent mechanisms at the TGN. We could not conduct an *in vitro* assay to show GSV formation from the TGN in *Gga1*kd C2C12 cells; therefore, it is currently unclear whether the defect in the insulin-dependent glucose uptake of *Gga1*kd is caused by a deficiency in the formation of functional GSVs.

According to previous biochemical approaches, GGA1 and GGA3, but not GGA2, were proposed to share certain functional and physiological features, such as their binding capacity to ubiquitin and their capability for self-inhibition [18]. Moreover, all three GGAs share similar structural features and binding affinity for the (D/E)xxLL-type signals of some membrane proteins [18]. Analysis of *Gga* KO mice also showed that single knock-out of *Gga1* or *Gga3* had no obvious phenotype, but that the *Gga1/Gga3* double knock-out, or *Gga2* single knock-out caused embryonic lethality, suggesting their diverse functional specificities *in vivo* [9,10]. In the current study, the result that GGA1, but not GGA2, was required for myotube formation in C2C12 cells, suggested that GGA1 might be responsible for the recognition of certain cargo molecules that are specifically required for myogenesis. Hence, the possible contribution of GGA3 to myogenesis is under investigation in our laboratory. In addition, our current approach using *in vivo* biotinylation for proximity labeling will provide clues to identify GGA1-specific interacting proteins that play important roles in myogenesis. Thus, further investigation into the specific cargo molecules associated with GGAs and their physiological analysis in skeletal muscle-specific *Gga1* KO mice will shed light on the functions of GGA clathrin adaptors in muscle tissue.

## Supporting information

S1 Fig GGA1 knockdown caused a defect in myotube formation. **(A)** C2C12 cells treated with control siRNA (a-d) or siRNA targeting *Gga*1 (e-h) were differentiated for 0, 2, 4 and 7 days. Phase contrast images (gray) merged with the Hoechst33342 images (blue) are shown. **(B)** Fusion index in (A) was calculated as described in Materials and Methods. Bar indicates 10 mm.
(TIF)

S2 Fig GGA1 knockdown affected the myoblast fusion. C2C12 cells treated with control siRNA (control) or siRNA targeting *Gga1* (GGA1 kd) were differentiated for 4 days and the population of the myotubes with the indicated number of nuclei was plotted.
(TIF)

S3 Fig Induction of IR expression in myotubes. Wild-type C2C12 was subjected to differentiation for 4 days, then indirect immunofluorescent microscopy was carried out. Images for IR expression (green; a and a’, c and c’), nuclear staining images with Hoechst33342 (blue: b and b’, c and c’) and merged images are shown. Multinuclear myotube (arrow) showed IR staining whereas little or no staining was seen in the mononuclear myoblasts (arrow heads).
(TIF)

S4 Fig *In vivo* proximity biotin labeling of IR by GGA1-BirA. To perform the *in vivo* proximal biotinylation, the ORF of GGA1was subcloned into pcDNA3.1 MCS-BirA(R118G), which was a gift from Dr. Kyle Roux (Addgene plasmid #336047)[35]. **(A)** pcDNA3.1 MCS-BirA(R118G) (BirA-HA) and pcDNA3.1-GGA1-BirA(R118G) (GGA1-BirA-HA) were transfected into C2C12 cells and intracellular localization of BirA-HA (a) and GGA1-BirA-HA (b) was examined by immunofluorescent microscopy with anti-HA antibody. The GGA1-BirA was localized at the perinuclear Gogi area (arrows) and at the peripheral puncta (arrow heads), whereas BirA alone did not localized at any intracellular compartments. **(B)** C2C12 cells were subjected to differentiation for 4 days and transfection of BirA-HA and GGA1-BirA-HA expression constructs was performed. Twenty-four hours after transfection, cells were incubated with 50 mM biotin for 6 hours and lysed. The biotinylated proteins were captured by streptavidin-sepharose (Wako). The input total lysate (5%) and the biotinylated proteins were subjected to immunoblotting with anti-IR antibody. The result indicated that GGA1-BirA-HA successfully biotinylated IR *in vivo*.
(TIF)
