# Correction: Clathrin adaptor GGA1 modulates myogenesis of C2C12 myoblasts

**DOI:** 10.1371/journal.pone.0209441

**Published:** 2018-12-13

**Authors:** Mari Isobe, Sachiko Lee, Satoshi Waguri, Satoshi Kametaka

A reference is omitted from the first sentence of the first paragraph under the subheading “GGA1 is involved in expression of the insulin receptor” in the Results section.

The sentence should read: Recently, it was shown that GGAs and the related clathrin adaptor AP-1 complex have functions not only in TGN-endosomal/lysosomal membrane trafficking, but also for the surface expression of a series of integral membrane proteins, including Notch and the epidermal growth factor receptor [13,21, Uemura et al., 2018].

The reference is also omitted from the last sentence of the first paragraph in the Discussion section.

The sentence should be read: Indeed, a recent report showed that GGA2 has a crucial role in the cell surface expression of the epidermal growth factor receptor (EGFR), and the GGA-related clathrin adaptor AP-1 complex is also involved in targeting of the cell surface signaling molecule Notch and the secretion of certain soluble factors [21, Uemura et al., 2018, 24,25].

Lastly, the reference is omitted from the first sentence of the 6th paragraph in the Discussion section.

The sentence should be read: More recently, Uemura and coworkers showed that GGA2 regulates the cell surface expression of EGFR via direct recognition and binding to the cytoplasmic region of EGFR [Uemura et al., 2018].

The reference is: Uemura T, Kametaka S, Waguri S. GGA2 interacts with EGFR cytoplasmic domain to stabilize the receptor expression and promote cell growth. Sci. Rep. 2018; 8: 1368. https://doi.org/10.1038/s41598-018-19542-4 PMID: 29358589

There is an also an error in reference 13. The correct reference is: Uemura T, Sawada N, Sakaba T, Kametaka S, Yamamoto M, Waguri S. Intracellular localization of GGA accessory protein p56 in cell lines and central nervous system neurons. Biomed. Res. 2018; 39: 179–187. https://doi.org/10.2220/biomedres.39.179 PMID: 30101838

There are a number of errors in the caption for Fig 4, “*Gga1* knockdown caused a defect in the expression of MYH3.” Please see the complete, correct Fig 4 caption here.

**Fig 4:**
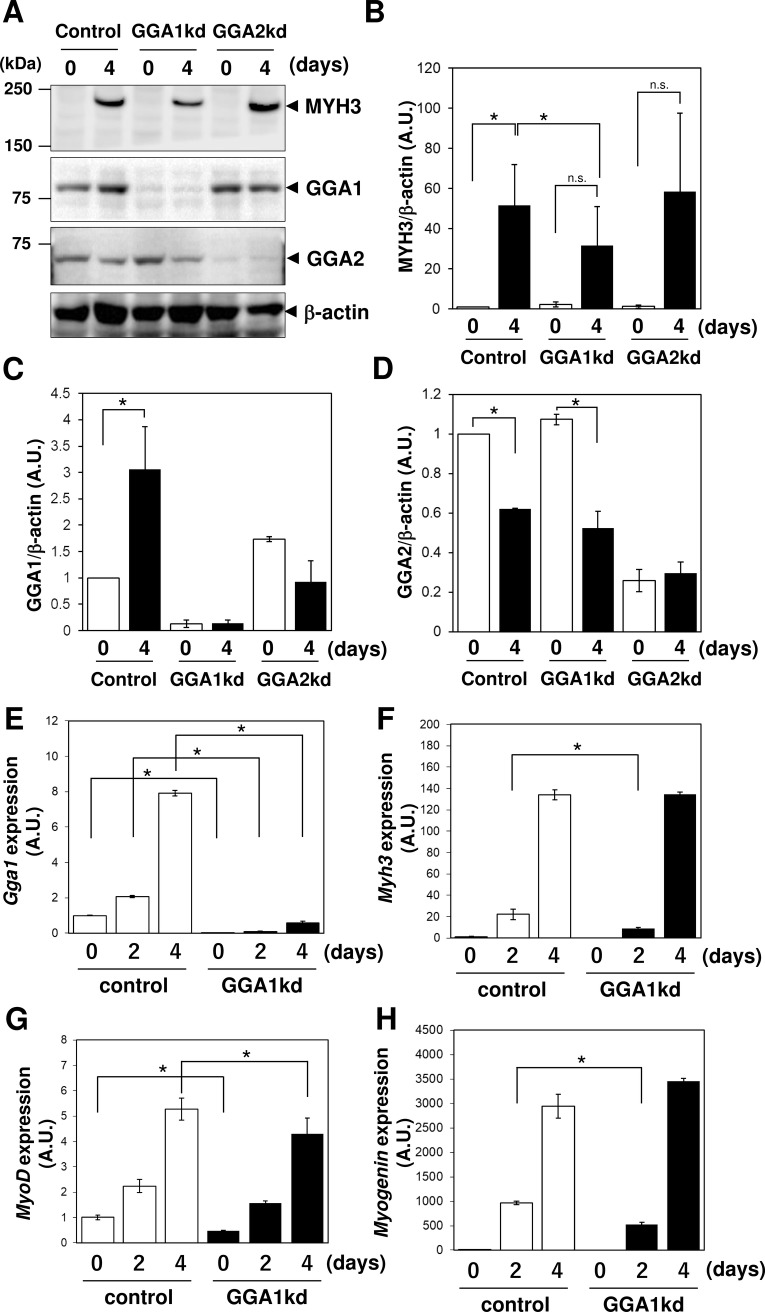
*Gga1* knockdown caused a defect in the expression of MYH3. (A) Golgi associated, gamma adaptin ear containing, ARF binding protein (GGA)1, GGA2 and myosin heavy chain 3 (MYH3) expression in control, *Gga1*kd and *Gga2*kd cells before (day0) and after (day4) myogenic differentiation were examined by immunoblotting. β-actin was used as a loading control. (B-D) Quantification of the expression level of each protein shown in (A). (E-H) Expression of *Gga1* gene and myogenic genes (*Myod* (myosin D), *Myog* (myogenin), and *Myh3*) at the differentiation periods of 0, 2 and 4 days in the control and *Gga1*kd cells was analyzed by qPCR. *Actβ* (β-actin) was used as the endogenous control. Error bars indicate SD (n = 3). *, *P* < 0.05 by Welch’s *t-*test.
