# Supplementary figures and images for: Clathrin adaptor GGA1 modulates myogenesis of C2C12 myoblasts
Source: PLoS One. 2018 Nov 15;13(11):e0207533. doi: 10.1371/journal.pone.0207533 (PMC6237421; doi:10.1371/journal.pone.0207533)

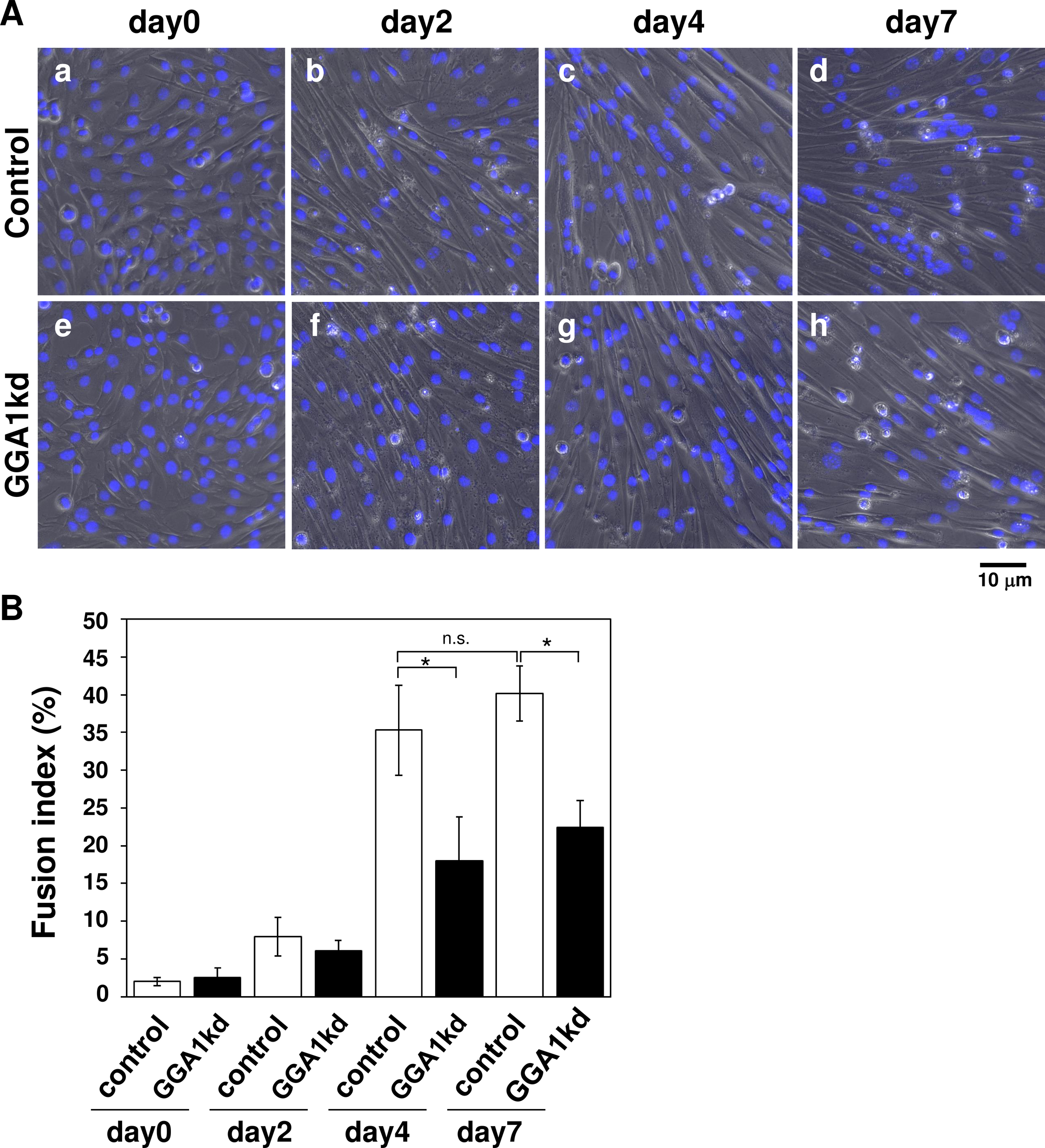

Supplement: S1 Fig — (A) C2C12 cells treated with control siRNA (a-d) or siRNA targeting Gga1 (e-h) were differentiated for 0, 2, 4 and 7 days. Phase contrast images (gray) merged with the Hoechst33342 images (blue) are shown. (B) Fusion index in (A) was calculated as described in Materials and Methods. Bar indicates 10 mm. (TIF) [file pone.0207533.s001.tif]

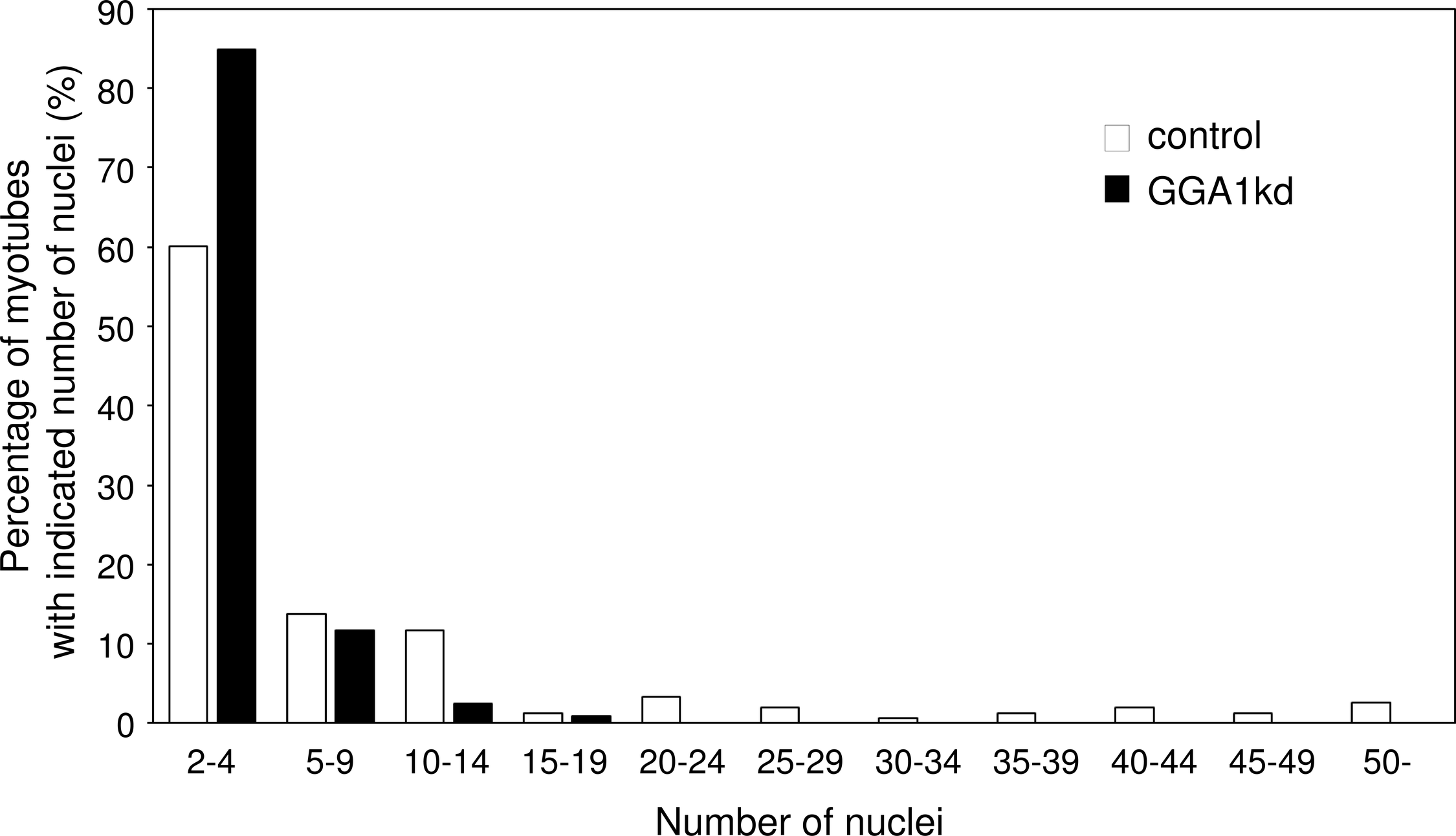

Supplement: S2 Fig — C2C12 cells treated with control siRNA (control) or siRNA targeting Gga1 (GGA1 kd) were differentiated for 4 days and the population of the myotubes with the indicated number of nuclei was plotted. (TIF) [file pone.0207533.s002.tif]

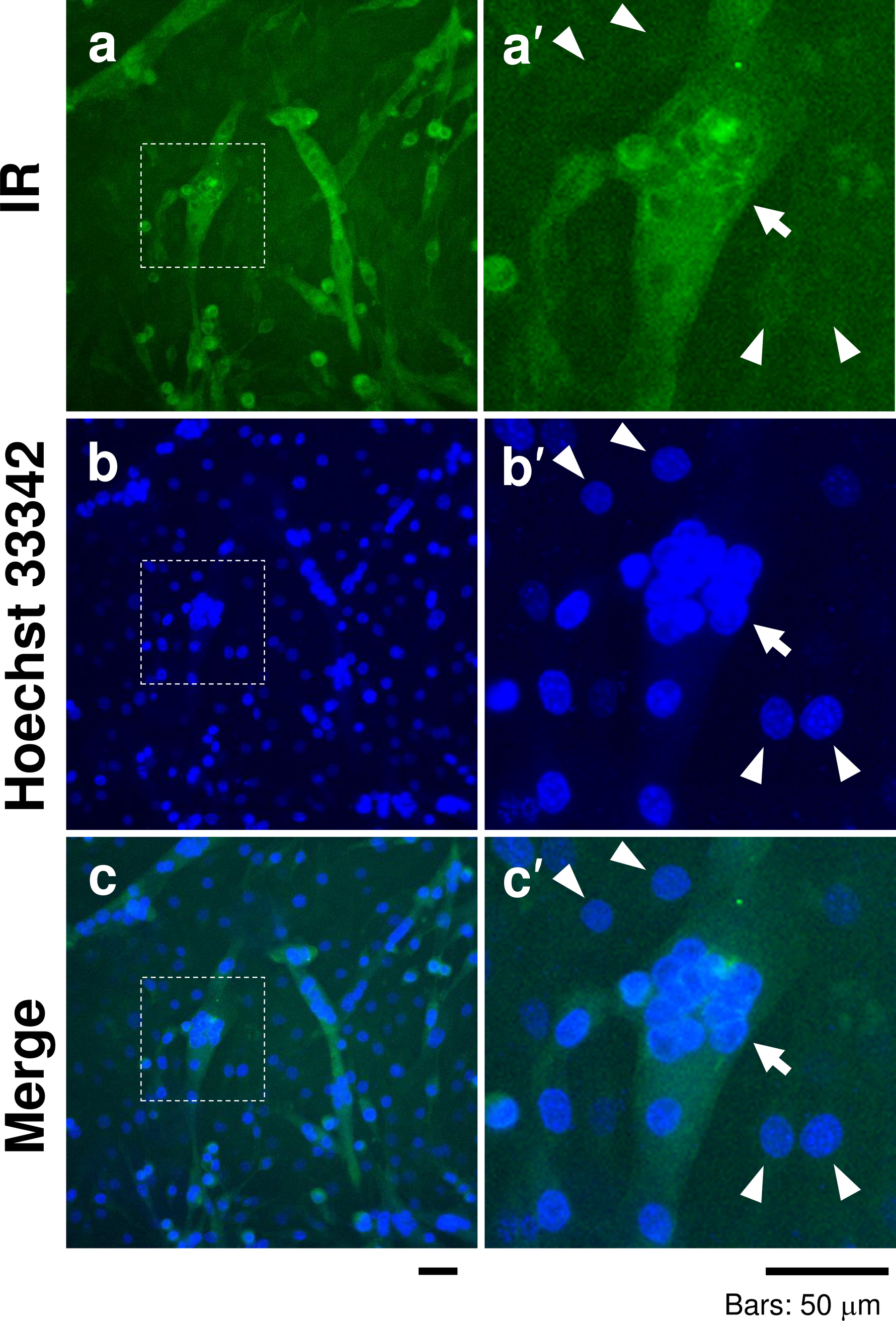

Supplement: S3 Fig — Wild-type C2C12 was subjected to differentiation for 4 days, then indirect immunofluorescent microscopy was carried out. Images for IR expression (green; a and a’, c and c’), nuclear staining images with Hoechst33342 (blue: b and b’, c and c’) and merged images are shown. Multinuclear myotube (arrow) showed IR staining whereas little or no staining was seen in the mononuclear myoblasts (arrow heads). (TIF) [file pone.0207533.s003.tif]

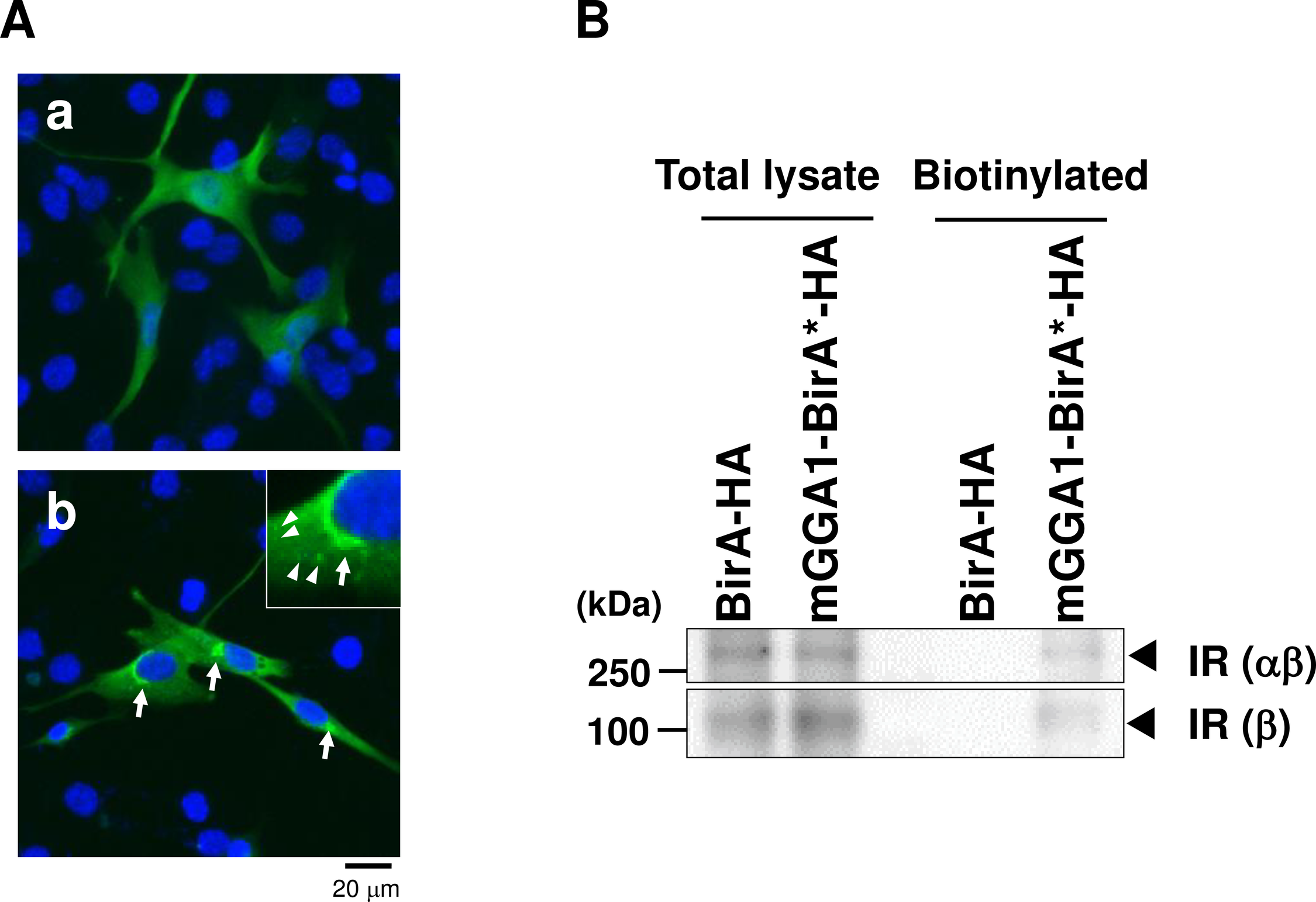

Supplement: S4 Fig — To perform the in vivo proximal biotinylation, the ORF of GGA1was subcloned into pcDNA3.1 MCS-BirA(R118G), which was a gift from Dr. Kyle Roux (Addgene plasmid #336047)[35]. (A) pcDNA3.1 MCS-BirA(R118G) (BirA-HA) and pcDNA3.1-GGA1-BirA(R118G) (GGA1-BirA-HA) were transfected into C2C12 cells and intracellular localization of BirA-HA (a) and GGA1-BirA-HA (b) was examined by immunofluorescent microscopy with anti-HA antibody. The GGA1-BirA was localized at the perinuclear Gogi area (arrows) and at the peripheral puncta (arrow heads), whereas BirA alone did not localized at any intracellular compartments. (B) C2C12 cells were subjected to differentiation for 4 days and transfection of BirA-HA and GGA1-BirA-HA expression constructs was performed. Twenty-four hours after transfection, cells were incubated with 50 mM biotin for 6 hours and lysed. The biotinylated proteins were captured by streptavidin-sepharose (Wako). The input total lysate (5%) and the biotinylated proteins were subjected to immunoblotting with anti-IR antibody. The result indicated that GGA1-BirA-HA successfully biotinylated IR in vivo. (TIF) [file pone.0207533.s004.tif]
